# Trends and Spatio-temporal variation of female genital mutilation among reproductive-age women in Ethiopia: a Spatio-temporal and multivariate decomposition analysis of Ethiopian demographic and health surveys

**DOI:** 10.1186/s12889-020-08882-4

**Published:** 2020-05-19

**Authors:** Getayeneh Antehunegn Tesema, Chilot Desta Agegnehu, Achamyeleh Birhanu Teshale, Adugnaw Zeleke Alem, Alemneh Mekuriaw Liyew, Yigizie Yeshaw, Sewnet Adem Kebede

**Affiliations:** 1 Department of Epidemiology and Biostatistics, Institute of Public Health, College of Medicine and Health Sciences, University of Gondar, Gondar, Ethiopia; 2 School of Nursing, College of Medicine and Health Sciences and Comprehensive specialized hospital, University of Gondar, Gondar, Ethiopia; 3 Department of Physiology, School of Medicine, College of Medicine and Health Sciences, University of Gondar, Gondar, Ethiopia

**Keywords:** Ethiopia, female genital mutilation, multivariate decomposition analysis, spatial analysis

## Abstract

**Background:**

Female genital mutilation (FGM) is a serious health problem globally with various health, social and psychological consequences for women. In Ethiopia, the prevalence of female genital mutilation varied across different regions of the country. Therefore, this study aimed to investigate the trend and determinants of female genital mutilation among reproductive-age women over time.

**Methods:**

A secondary data analysis was done using 2000, 2005, and 2016 Demographic Health Surveys (DHSs) of Ethiopia. A total weighted sample of 36,685 reproductive-age women was included for analysis from these three EDHS Surveys. Logit based multivariate decomposition analysis was employed for identifying factors contributing to the decrease in FGM over time. The Bernoulli model was fitted using spatial scan statistics version 9.6 to identify hotspot areas of FGM, and ArcGIS version 10.6 was applied to explore the spatial distribution FGM across the country.

**Results:**

The trends of FGM practice has been decreased from 79.9% in 2000 to 70.4% in 2016 with an annual reduction rate of 0.8%. The multivariate decomposition analysis revealed that about 95% of the overall decrease in FGM practice from 2000 to 2016 was due to the difference in the effects of women’s characteristics between the surveys. The difference in the effects of residence, religion, occupation, education, and media exposure were significant predictors that contributed to the decrease in FGM over time. The spatial distribution of FGM showed variation across the country. The SaTScan analysis identified significant hotspot areas of FGM in Somali, Harari, and Afar regions consistently over the three surveys.

**Conclusion:**

Female genital mutilation practice has shown a remarkable decrease over time in Ethiopia. Public health programs targeting rural, non-educated, unemployed, and those women with no access to media would be helpful to maintain the decreasing trend of FGM practice. The significant Spatio-temporal clustering of FGM was observed across regions in Ethiopia. Public health interventions must target the identified clusters as well.

## Background

Female Genital Mutilation (FGM) is a partial or total removal of the external female genitalia or other injuries to the female genital organs for non-medical reasons [1]. Globally, more than 200 million girls and women undergo female genital mutilation, of these, 125 million were practiced in Africa [2]. It is widely practiced in Asia, Middle East, South America, and Africa [3], particularly in the north-eastern regions of Africa: Djibouti, Eritrea, Ethiopia, and Somalia [4]. Nowadays, the trends of FGM have been reduced dramatically in Africa [5] and it varies greatly across countries over time [6, 7]. For example, the prevalence of FGM has reduced from 71.4% in 1995 to 8% in 2016 in East Africa [6].

The Ethiopian government has prioritized sustainable development goal 5.3 as one of the national development targets to eliminate FGM [8, 9]. Due to the establishment of this goal, the practice of FGM has been decreased from 79.9 to 65% from 2000 to 2016 in Ethiopia [10, 11]. Despite this progress, still, around 23.8 million girls had undergone FGM, making the second African country in FGM [12].

FGM is associated with an increased risk of both short and long term health problems [13]. It poses serious physical, psychological and health consequences to women [14]. It also increases the risk of complications during pregnancy and childbirth, obstetric fistula, sexual problem, menstrual abnormality, post-traumatic stress disorder, recurrent pelvic, and vaginal infection [15].

Though, the prevalence of FGM has been varied across regions in Ethiopia [16–18], previous studies in this aspect focused on the prevalence and associated factors of FGM only [16, 17, 19–22] and failed to capture the trend and Spatio-temporal variation of FGM over time. Thus, the identification of geographic areas with a high prevalence of FGM has become indispensable to design targeted interventions. Therefore, this study aimed to investigate the trend and determinants of FGM among reproductive-age women in Ethiopia.

## Methods

### Data sources and sampling procedures

**Fig. 1:**
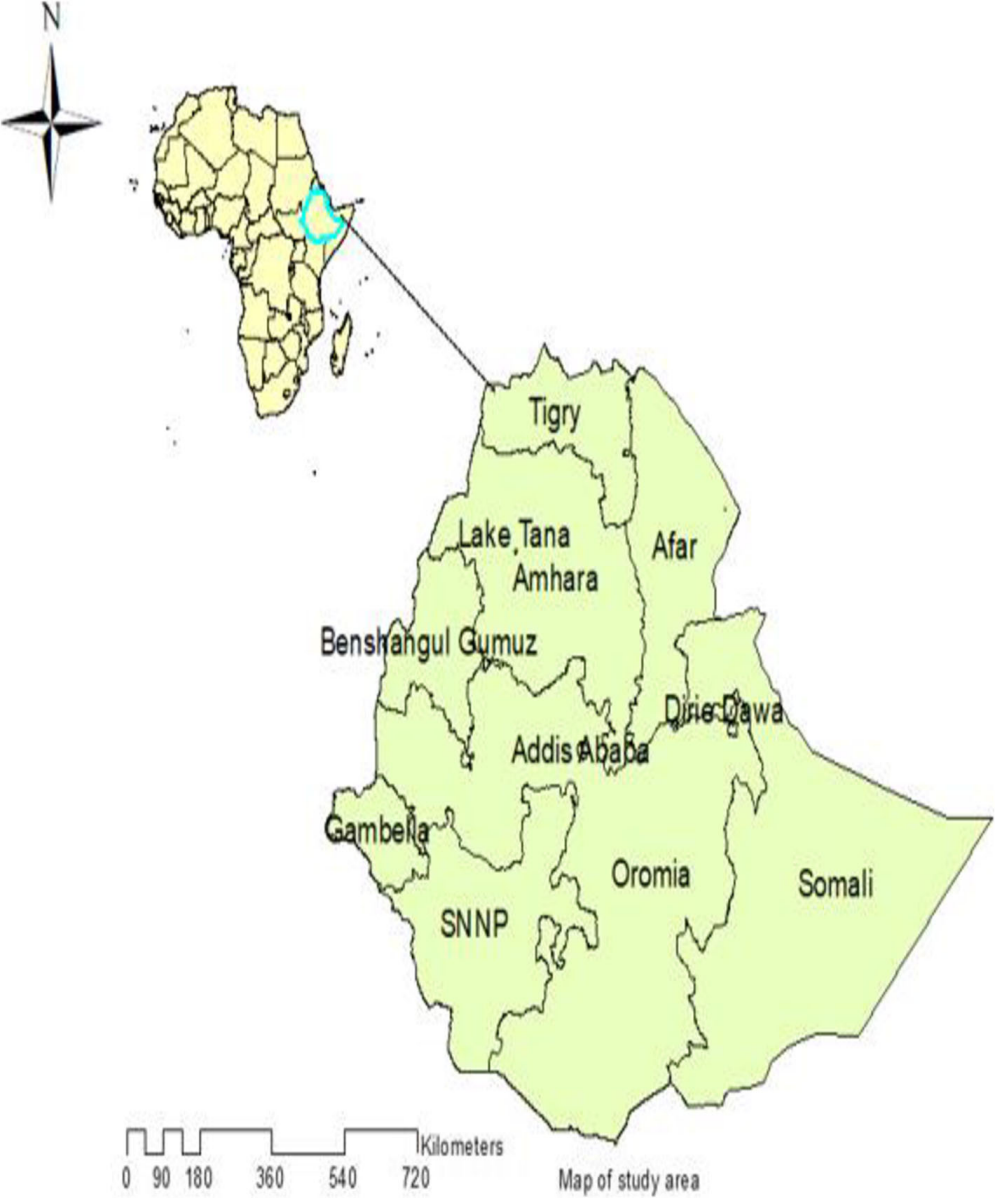
Map of the study area (Source; Shape file from CSA, 2013, done using ArcGIS version 10.6 and SaTScan version 9.6)

We used 2000, 2005, and 2016 Ethiopian Demographic and Health surveys (EDHSs). These EDHSs are nationally representative cross-sectional surveys performed in 9 regions and 2 country city administrations every five years (Fig. 1). In each of the surveys, stratified two-stage sampling of clusters was carried out. Stratification was achieved by separating each region into urban and rural areas. Accordingly, a total of 21 sampling strata have been created. In the first stage, a total of 539 Enumeration Areas (EAs) for EDHS 2000, 540 EAs for EDHS 2005, and 645 EAs for EDHS 2016 were randomly selected proportional to the EA size. At the second stage, on average 27 to 32 households per EA were selected. A total weighted sample of 36,685 (15,367 in EDHS 2000, 14,070 in EDHS 2005 and 7248 in EDHS 2016) reproductive-age women used for this study. The comprehensive procedure for sampling was described in the complete EDHS report [10, 11, 23].

### Study variables

The outcome variable for this study was experienced FGM and coded as “Yes = 1” or “No = 0”. The EDHS asked women to answer the question “have you ever been circumcised?”. So, the response variable of the ith mother Yi was measured as a dichotomous variable with possible values Yi = Yes if ith mother had experienced circumcision and Yi = No if mother did not experience circumcision. The independent variables included in this study were: residence, religion, geographic region, responded age, maternal education, women occupation, media exposure, and wealth index.

### Statistical analysis

The data were extracted from the Individual Record (IR) data sets. Before any statistical analysis, the data were weighted using sampling weight, primary sampling unit, and strata, to restore the representativeness of the survey and get reliable statistical estimates.

### Trend analysis

Trend analysis of FGM and decomposition of the decrease in the prevalence of FGM over time was done. The trend analysis has been done in three phases, phase 1 (2000–2005), phase 2 (2005–2016) and the overall trend (2000–2016), the trend and determinants was examined separately.

For the trend analysis multivariate decomposition analysis for non-linear response outcome was employed to identify the factors contributed to the decrease in FGM practice across the surveys. For our study, Logit based decomposition analysis was employed.

The Logit based multivariate decomposition analysis utilizes the output from the logistic regression model to parcel out the observed decrease in FGM over time into components. The main aim of multivariate decomposition is to identify the factors contributing to the decrease in FGM practice for the last 16 years. The decrease in FGM practice can be explained by the compositional difference between surveys (i.e. differences in characteristics) and/or the difference in effects of explanatory variables (i.e. differences in the coefficients) between the surveys. Hence, the observed decrease in FGM over time is additively decomposed into a characteristics (or endowments) component and a coefficient (or effects of characteristics) component.

For logistic regression, the Logit or log-odd of FGM is taken as:

$$ \mathrm{Logit}\ \left(\mathrm{A}\right)-\mathrm{Logit}\ \left(\mathrm{B}\right)=\mathrm{F}\ \left(\mathrm{XA}\upbeta \mathrm{A}\right)-\mathrm{F}\ \left(\mathrm{XB}\upbeta \mathrm{B}\right) $$

$$ =\left[\mathrm{F}\ \left(\mathrm{XA}\upbeta \mathrm{A}\right)-\mathrm{F}\ \left(\mathrm{XB}\upbeta \mathrm{A}\right)\right]+\left[\mathrm{F}\ \left(\mathrm{XB}\upbeta \mathrm{A}\right)-\mathrm{F}\ \right(\mathrm{XB}\upbeta \mathrm{B}\Big] $$

E C

The E component refers to the part of the differential owing to differences in endowments or characteristics. The C component refers to that part of the differential attributable to differences in coefficients or effects.

The recently developed multivariate decomposition for the non-linear model was used for the decomposition analysis of female genital mutilation using the mvdcmp STATA command [24].

### Spatial analysis

ArcGIS version 10.6 and SaTScan version 9.6 software were used for spatial analysis. The spatial autocorrelation (Global Moran’s I) statistic was used to assess whether there was significant clustering of FGM [25]. Moran’s I has a value ranging from-1 to 1. Positive Moran’s I value shows that FGM is clustered while negative Moran’s I indicates that FGM is dispersed [26]. The value of Moran’s I near zero has revealed that FGM is randomly distributed. Both Z-score and *P*-value are generated to assess the significance of the Moran index.

In spatial scan statistical analysis, Bernoulli based model was employed to identify significant spatial high FGM clusters using Kuldorff’s SaTScan version 9.6 software. The SaTScan uses a circular scanning window that moves across the study area. Women who were circumcised were taken as cases whereas those who were not circumcised were taken as controls to fit the Bernoulli model. The default maximum spatial cluster size of < 50% of the population was used as an upper limit, which allowed both small and large clusters to be detected and ignored clusters that contained more than the maximum limit. For each potential cluster, a likelihood ratio test statistic and the *p*-value were used to determine significant clusters. The scanning window with maximum likelihood was the most likely performing cluster. The primary and secondary clusters were identified and ranked based on their likelihood ratio test, based on 999 Monte Carlo replications [27].

The Ordinary Kriging spatial interpolation method was used to predict the un-sampled/unmeasured values from the sampled measurements.

### Ethical approval and consent to participate

Since the study was a secondary data analysis of publically available survey data from MEASURE DHS program, ethical approval and participant consent were not necessary for this particular study. We requested DHS Program and permission was granted to download and use the data for this study from http://www.dhsprogram.com. There are no names of individuals or household addresses in the data files. The geographic identifiers only go down to the regional level (where regions are typically very large geographical areas encompassing several states/provinces. In surveys that collect GIS coordinates in the field, the coordinates are only for the enumeration area (EA) as a whole, and not for individual households, and the measured coordinates are randomly displaced within a large geographic area so that specific enumeration areas cannot be identified.

## Results

### Characteristics of the study population

About one-third of the respondents in all three surveys were found in the Oromia region. There was a slight increment in urban residents from 18.2% in 2000 to 23.0% in 2016. In 2000 EDHS, 75.2% of women were not educated, while this figure decreased to 47.0% in 2016. The proportion of women who had media exposure has been increased from 34% in 2000 to 43.3% in 2016. Except for religion, maternal age, region, and wealth index, all other variables showed changes in the composition of women across the surveys (Table 1).

**Table 1:** Percentage distribution of characteristics of women in 2000, 2005 and 2016 Ethiopian Demographic and Health Surveys

Variables	EDHS 2000 (*N* = 15,367) Percentage (%)	EDHS 2005 (*N* = 14,070) Percentage (%)	EDHS 2016 (*N* = 7248) Percentage (%)
**Region**			
Tigray	6.3	6.5	6.4
Afar	1.2	1.0	0.9
Amhara	24.9	24.7	23.5
Oromia	38.6	35.6	37.9
Somali	1.1	3.5	3.2
Benishangul	1.0	0.9	0.9
SNNPRs	21.4	21.3	19.8
Gambella	0.3	0.3	0.2
Harari	0.3	0.3	0.3
Addis Ababa	4.4	5.4	6.3
Dire Dawa	0.5	0.5	0.6
**Place of residence**			
Urban	18.2	17.8	23.0
Rural	81.8	82.2	77.0
**Religion**			
Orthodox	50.5	49.2	43.6
Muslim	29.0	28.5	31.6
Catholic	1.1	1.3	0.7
Protestant	15.8	18.9	23.1
Traditional	3.3	1.3	0.8
Others	0.2	0.9	0.3
**Age of respondent (in years)**			
15–19	24.1	23.2	21.0
20–24	18.6	18.1	16.5
25–29	16.8	17.9	18.7
30–34	12.0	12.9	15.5
35–39	11.2	11.4	12.9
40–44	9.2	8.4	8.4
45–49	8.2	8.1	6.9
**Maternal education**			
No education	75.2	65.9	47.0
Primary	15.8	22.2	34.5
Secondary	8.5	10.5	12.3
Higher	0.5	1.4	6.2
**Maternal occupation**			
Not working	36.9	66.2	48.5
Professional	0.7	1.3	2.7
Clerical	0.6	0.4	1.0
Sales	13.3	10.6	15.6
Agriculture employee	37.0	17.7	20.6
Services	1.3	0.1	3.8
Skilled manual	9.6)	2.1	3.9
Unskilled manual	0.7	1.7	3.9
**Media exposure**			
Yes	34.0	46.3	43.3
No	66.0	53.7	56.7

### Trends of female genital mutilation

The overall FGM has been decreased from 79.9% (95% CI: 79.3, 80.5) in 2000 to 74.3% (95% CI: 73.5, 74.9) in 2005 to 70.4% (95% CI: 69.3, 71.4) in 2016 with annual reduction rate of 0.8%. The trends in female genital mutilation have shown variation by women’s characteristics over time. Major decreases in FGM were observed in some of the selected variables.

**Fig. 2:**
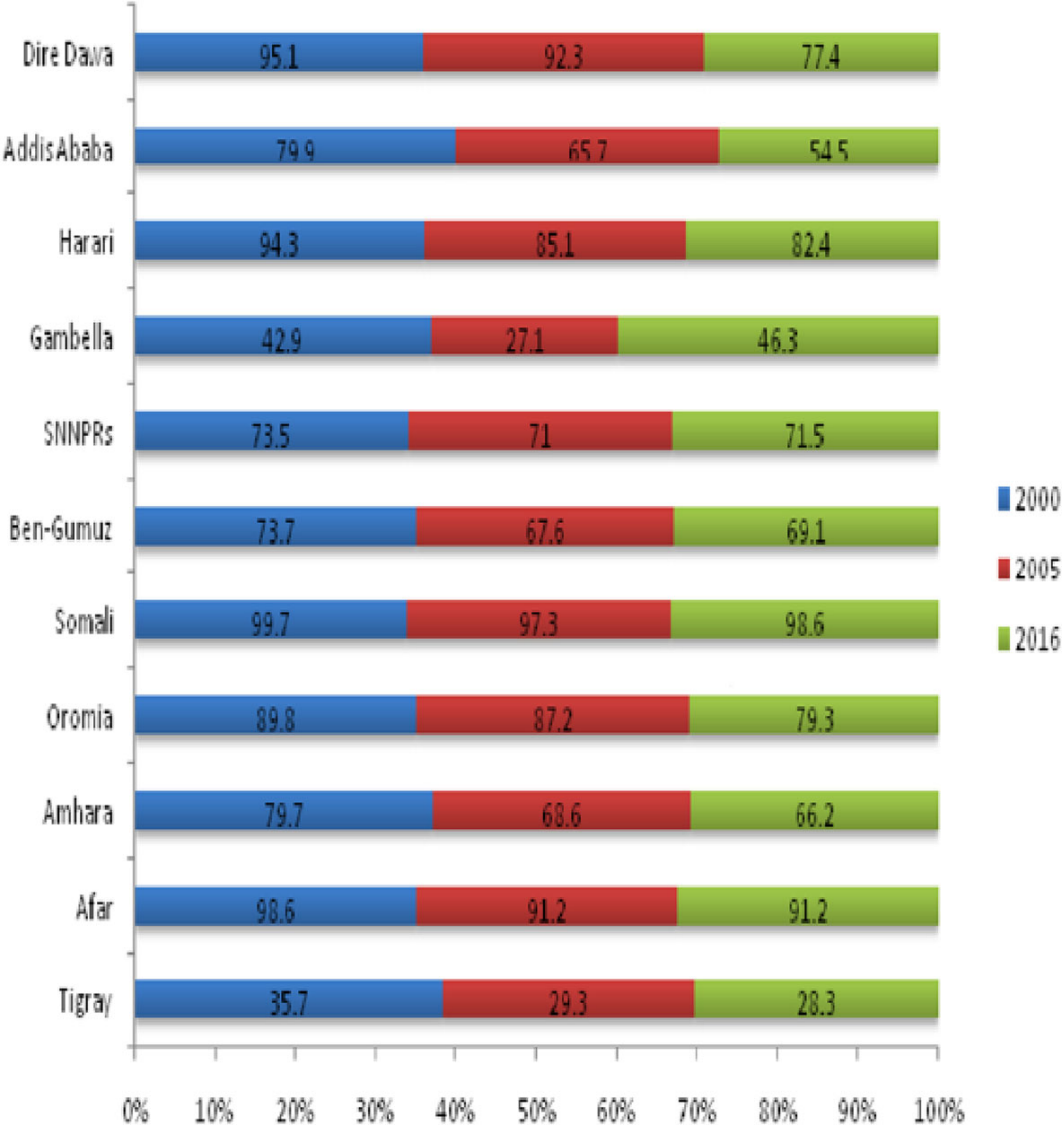
Trends of FGM practice among reproductive-age women across regions in Ethiopia, based on EDHS 2000, 2005 and 2016

Tigray, Harari, Addis Ababa, and Dire-Dawa regions showed the greatest decline in FGM practice over the study period but the prevalence remains high in the Somali region (Fig. 2). Among urban residents, the largest decline in FGM practice has been observed during the third phase of the study period (2000–2016), at 24.3 percentage point decrease as compared with 11.3 percentage point decrease during the first phase (2000–2005) and a 13.0 percentage point decrease during the second phase (2005–2016). Young age women (15–19 years) showed a larger decrease in FGM by 19.1 percentage point change during the overall study period (2000–2016). Women with secondary and higher education showed a 34.8 percentage point decrease during the third phase of the study period (2000–2016) (Table 2).

**Table 2:** The trend in FGM practice among reproductive-age women by selected characteristics based on 2000, 2005, and 2016 Ethiopian Demographic and Health Surveys

Characteristics	EDHS 2000	EDHS 2005	EDHS 2016	Point difference in the prevalence of FGM		
				Phase 1 (2005–2000)	Phase 2 (2016–2005)	Phase 3 (2016–2000)
**Place of residence**						
Urban	79.8	68.5	55.5	−11.3	−13.0	−24.3
Rural	79.9	75.5	74.8	−4.4	−0.7	−5.1
**Religion**						
Orthodox	76.8	67.5	58.8	−9.5	−8.7	−18.0
Muslim	91.8	88.9	84.9	−2.9	−4.0	−6.9
Catholic	66.9	76.9	73.7	10.0	−3.2	6.8
Protestant	71.9	71.3	73.2	−0.6	1.9	2.5
Traditional	66.8	49.2	62.1	−17.6	11.9	−4.7
**Age of women (in years)**						
15–19	70.7	62.2	51.6	−8.5	−10.6	−19.1
20–24	78.3	73.0	63.3	−5.3	−9.7	−15.0
25–29	81.4	77.6	73.4	−3.8	−4.2	−8.0
30–34	86.2	78.0	82.2	−8.2	4.2	−4.0
35–39	83.6	81.2	80.8	−2.4	−0.4	−2.8
40–44	85.8	81.6	77.2	−4.2	−4.4	−8.6
45–49	86.8	80.8	81.5	−6.0	0.7	−5.3
**Maternal education**						
No education	80.4	77.3	81.0	−3.1	3.7	0.6
Primary	78.4	70.8	66.3	−7.6	−4.5	−12.1
Secondary	77.7	65.1	50.7	−12.6	−14.4	−27.0
Higher	85.5	55.6	50.7	−29.9	−4.9	−34.8
**Women occupation**						
Not working	79.6	74.2	72.9	−5.4	−1.3	−6.7
Professional	85.5	65.2	61.9	−20.3	−3.3	− 23.6
Clerical	73.5	65.8	44.8	−7.7	−21.0	−28.7
Sales	83.8	74.6	69.6	−9.2	−5.0	−14.2
Agricultural employee	77.6	78.0	70.5	0.4	−7.5	−7.1
Services	86.2	67.5	72.2	−18.7	4.7	−14.0
Skilled manual	83.9	68.1	63.0	−15.8	−5.1	−20.9
Unskilled manual	80.6	55.9	58.2	−24.7	2.3	−22.4
**Media exposure**						
Yes	80.5	72.8	62.9	−7.7	−9.9	−17.6
No	79.7	75.5	76.5	−4.2	1.0	−3.2

### Decomposition analysis

The overall decomposition analysis revealed that about 95% of the overall decrease in FGM among reproductive-age women was due to difference in coefficient (effects of characteristics) across the surveys but the change due to the difference in composition (endowment) was not significant (Table 3). After controlling for the roles of compositional changes, a significant decrease in FGM practice was due to the difference in the effects of residence, region, educational status, religion, occupation, and media exposure. About 20% of the decrease in FGM practice over the last 16 years was attributable to the difference in the effect among the urban population (*B* = 0.02, 95% CI: 0.01, 0.03). As compared to the Somali region, the change in the effect of FGM practice among the Tigray regions (*B = 0*.01, 95% CI: 0.0008, 0.02) contributes about 12.2% of the overall decrease in FGM over the study period. Other factors such as a decrease in the effects of women higher education (*B* = -0.0008, 95% CI: − 0.001, − 0.00006), Muslim religion (*B* = − 0.05, 95% CI: − 0.08, − 0.01), Orthodox religion (*B* = -0.06, 95% CI: − 0.11, − 0.02), media exposure (*B* = -0.012, 95% CI: − 0.022, − 0.003) and occupation (*B* = -0.02, 95% CI: − 0.03, − 0.009) were significant predictors of the decrease in FGM practice over the last 16 years (Table 4).

**Table 3:** The overall decomposition analysis of the decrease in FGM practice among reproductive-age women in Ethiopia, 2000 to 2016

Female genital mutilation	Coef.	95% confidence interval	Pct.
E	−0.0053	(−0.014, 0.0037)	5.1
C	−0.099	(− 0.114, − 0.084)^**^	94.9
R	− 0.104	(− 0.115, − 0.093)	

^**^P-value < 0.01, E: Endowment, C: Coefficient, R: Residual, Coef: Coefficient, pct.: Percentage

**Table 4:** The detailed decomposition analysis of the change in FGM practice in Ethiopia, 2000 to 2016

Female Genital Mutilation		Difference due to characteristics (E)		Difference due to coefficient (C)	
		Coef.	Pct.	Coef.	Pct.
Residence	Rural	0		0	
	Urban	0.003 (− 0.006, 0.011)	−2.7	0.02 [0.01, 0.03]**	−20.6
Region	Somali	0		0	
	Afar	0.003 [− 0.006, 0.012]	−2.8	− 0.0006[− 0.01, 0.009]	0.6
	Amhara	−0.005 [− 0.02, 0.01]	4.9	0.013 [− 0.005, 0.03]	−12.0
	Oromia	−0.015 [− 0.06, − 0.029]	14.1	0.006 [− 0.02, 0.03]	−0.5
	Tigray	0.013 [−0.025, 0.05]	− 12.2	0.01 [0.0008, 0.02]**	− 12.2
	Ben-Gumuz	0.0029 [−0.0058, 0.012]	−2.8	0.007 [−0.002, 0.016]	−6.5
	SNNPRs	−0.0046[− 0.018, − 0.009]	4.4	0.01 [− 0.009, 0.029]	−9.7
	Gambella	0.004 [− 0.009, 0.017]	−4.1	0.007 [− 0.0008, 0.015]	− 6.9
	Harari	−0.0004 [− 0.002, 0.0008]	0.37	0.003 [− 0.006, 0.011]	−2.4
	Addis Ababa	−0.005 [− 0.02, 0.01]	4.9	0.011 [− 0.007, 0.03]	−11.2
	Dire Dawa	0.001 [− 0.002, 0.004]	−1.0	−0.0013 [− 0.011, 0.009]	1.2
Women age (in years)	15–19	−0.0018 [− 0.007, 0.004]	1.7	−0.007 [− 0.015,0.0009]	6.7
	20–24	−0.0001[− 0.00002, 0.00001]	0.005	−0.002 [− 0.009, 0.004]	2.3
	25–29	0.0003 [−0.0006, 0.0012]	− 0.28	−0.002 [− 0.008, 0.0035]	2.4
	30–34	0.0001 [−0.0006, 0.001]	− 0.1	0.0007 [− 0.004, 0.005]	−0.6
	35–39	0		0	
	40–44	−0.0001 [− 0.0003, 0.0002]	0.07	− 0.001 [− 0.005, 0.002]	1.2
	45–49	00002 [− 0.0008, 0.0013]	−0.2	0.0006 [− 0.003, 0.004]	−0.5
Educational status	No education	0		0	
	Primary	0.0019[−0.007, 0.01]	−1.8	−0.002 [− 0.006, 0.002]	1.8
	Secondary	0.0005 [−0.001, 0.002]	−0.5	− 0.001 [− 0.006, 0.003]	1.2
	Higher	0.008 [−0.02, 0.033]	−7.6	− 0.0008 [− 0.001, − 0.00006]**	0.7
Occupation	Not working	0		0	
	Gov’t employee	−0.0013 [− 0.0012, 0.0009]	0.1	− 0.0006 [− 0.001, 0.0003]	0.5
	Self -employee	0.0007 [− 0.003, 0.005]	0.7	−0.02 [− 0.03, − 0.009]**	19.7
Religion	Orthodox	− 0.0018 [− 0.011, 0.007]	1.8	−0.06 [− 0.11, − 0.02]**	61.9
	Muslim	− 0.006 [− 0.025,0.012]	5.8	−0.05 [− 0.08, − 0.01]**	43.6
	Protestant	0.0007 [− 0.0043, 0.0056]	−0.6	− 0.003 [− 0.016, 0.01]	3.1
	Traditional	−0.0038 [− 0.015, 0.0075]	3.6	−0.002 [− 0.006, 0.002]	1.5
	Catholic	0		0	
Media exposure	Yes	−0.0006 [− 0.0025,0.0014]	0.5	− 0.012 [− 0.022, − 0.003]**	11.8
	No	0		0	
Constant				0.02 [−0.15, 0.20]	−23.4

^**^P-value < 0.01, Coef: Coefficient, Pct.: Percentage

### Spatial distribution of FGM practice

**Fig. 3:**
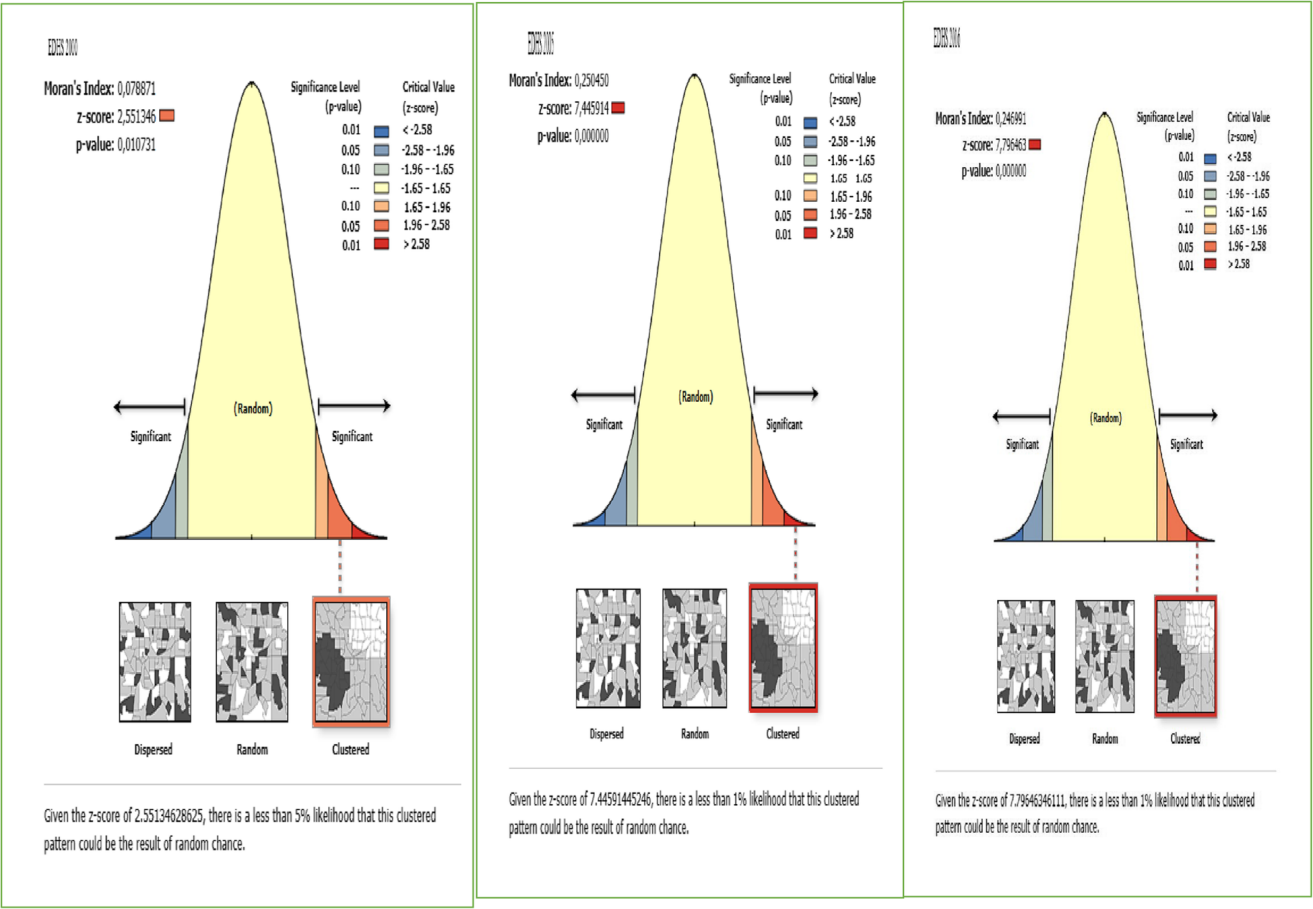
Global Spatial autocorrelation of FGM in Ethiopia, 2000, 2005 and 2016

**Fig. 4:**
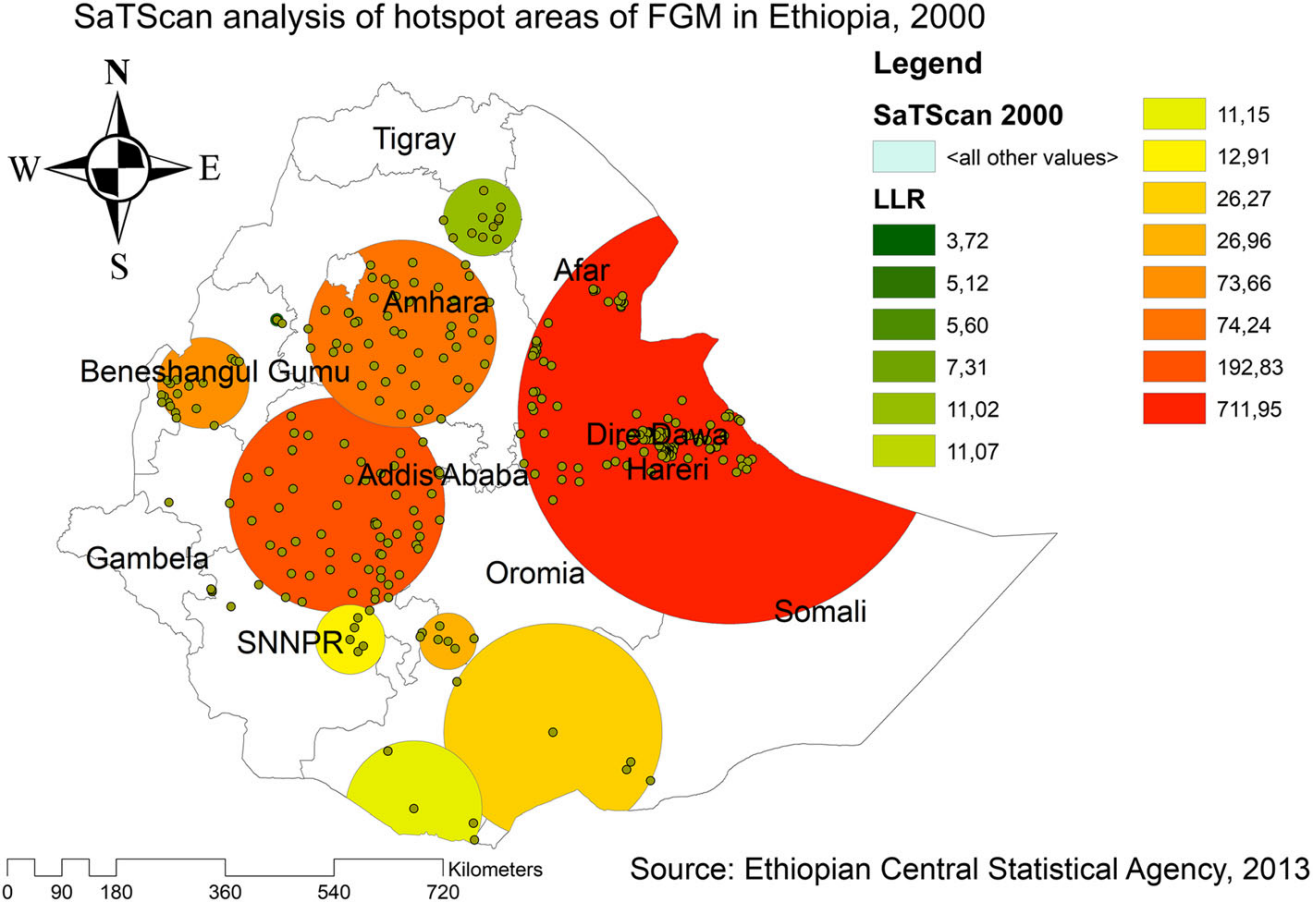
SaTScan analysis of FGM practice in Ethiopia, 2000 (Source; Shape file from CSA, 2013, done using ArcGIS version 10.6 and SaTScan version 9.6)

The spatial distribution of FGM showed significant spatial variations across the country (Fig. 3). The highest prevalence of FGM was identified in the Somali, Harari, east Benishangul Gumuz, Amhara, Oromia, east SNNPRs, and southern Afar regions consistently over time. Whereas, Gambella, Tigray, northeast Amhara, west SNNPRs and northeast Benishangul Gumuz regions showed the lowest FGM. In EDHS 2000, the spatial scan statistics identified 427 significant primary and secondary clusters. Of these, 141 clusters were the most likely clusters (primary clusters), which was located in north Somali, Harari, Dire Dawa, south Afar, and southeast Amhara region centered at 9.867651 N, 43.086403 E with 348.25 km radius, a Relative Risk (RR) of 1.35, and Log-Likelihood Ratio (LRR) of 711.95, at *p*-value< 0.001. It showed that women within the spatial window had 1.35 times higher odds of having FGM as compared to women outside the spatial window, whereas the secondary clusters were located in Amhara, Tigray, Benishangul Gumuz, SNNPRs, and Oromia regions (Fig. 4).

**Fig. 5:**
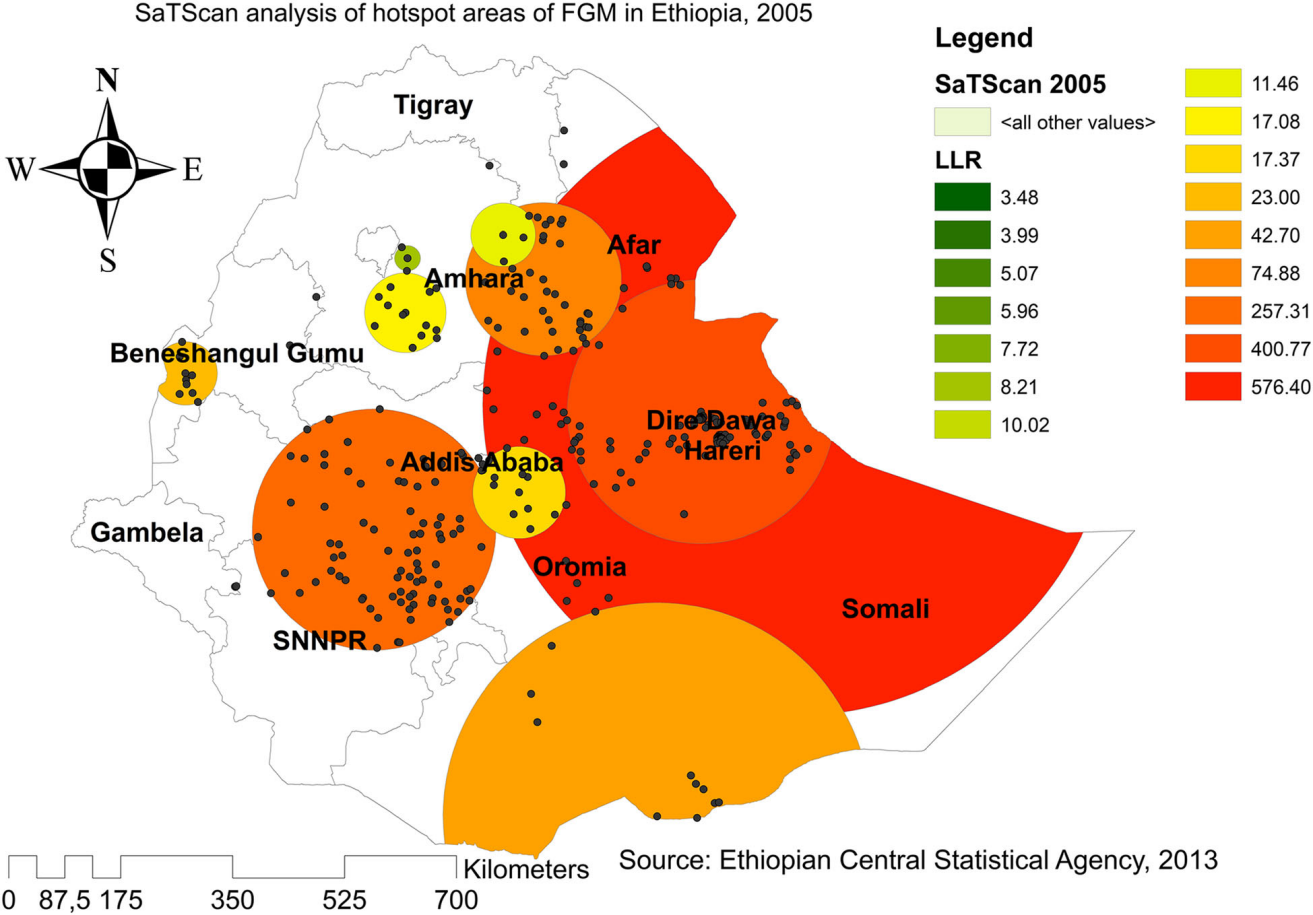
SaTScan analysis of FGM practice in Ethiopia, 2005 (Source; Shape file from CSA, 2013, done using ArcGIS version 10.6 and SaTScan version 9.6)

**Fig. 6:**
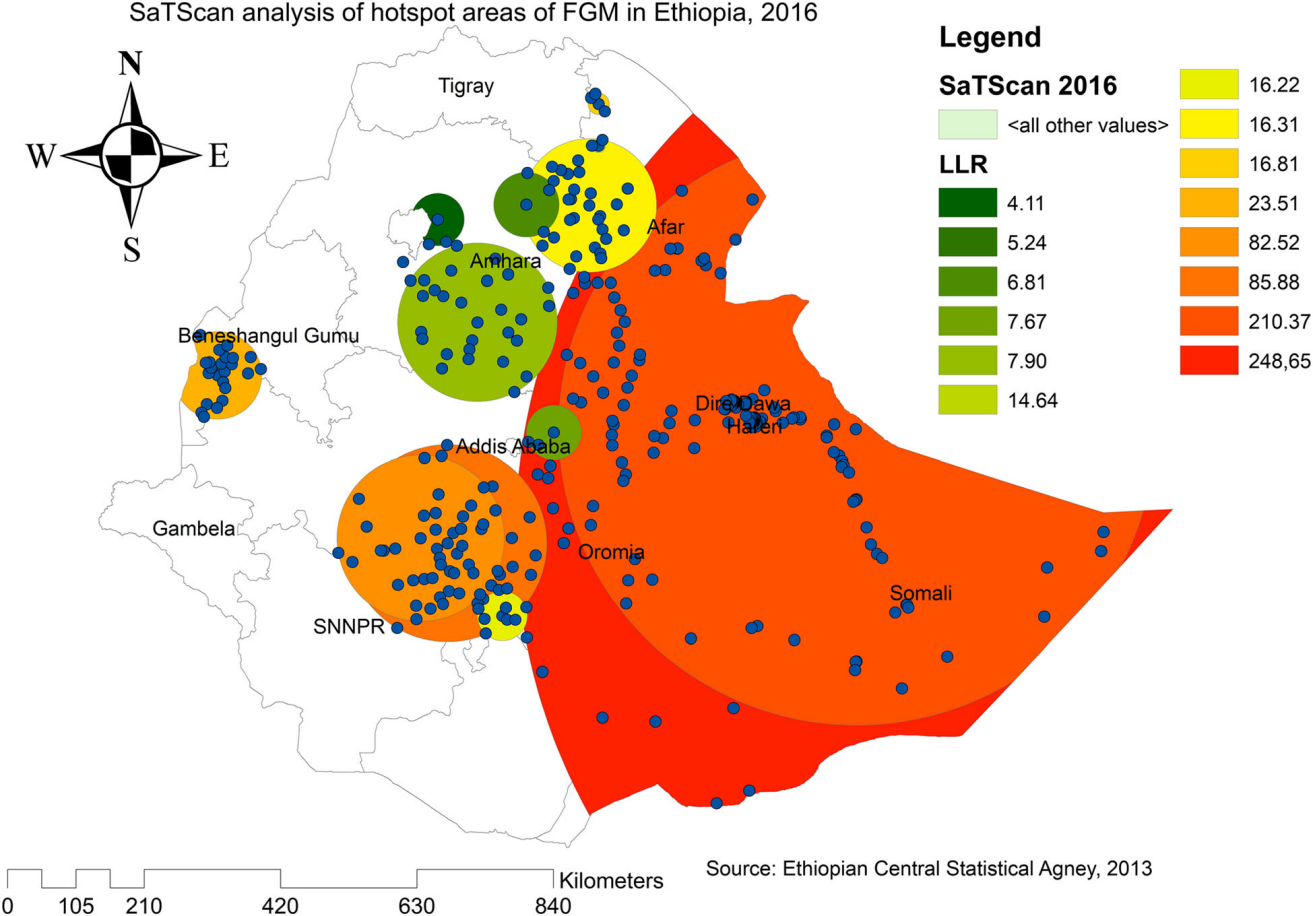
SaTScan analysis of FGM practice in Ethiopia, 2016 (Source; Shape file from CSA, 2013, done using ArcGIS version 10.6 and SaTScan version 9.6)

In EDHS 2005, a spatial scan statistics identified a total of 468 significant primary and secondary clusters. Of these, 175 clusters were the most likely clusters (primary clusters), which was located in the entire Somali region and border areas of Somali regions, centered at 9.774395 N, 43.208576 E with a radius of 484.13 km, a Relative Risk (RR) of 1.42, and Log-likelihood (LLR = 576.40, p-value< 0.0001). It showed that women within the spatial window had 1.42 times higher odds of FGM as compared to women outside the spatial window and the secondary clusters were located in Benishangul, Amhara, Oromia, and Gambella regions (Fig. 5). In EDHS 2016, a spatial scan statistics identified a total of 581 significant primary and secondary clusters. Of these, 220 clusters were the most likely clusters (primary clusters) and the spatial window was located in the entire Somali, Afar and border areas of Somali, centered at 7.717178 N, 46.991580 E with a radius of 900.49 km, a Relative Risk (RR) of 1.42, and Log-likelihood (LLR = 248.65, p-value< 0.0001). It showed that women within the spatial window had 1.42 times higher odds of having circumcised as compared to women outside the spatial window (Fig. 6).

### Kriging interpolation of FGM practice

**Fig. 7:**
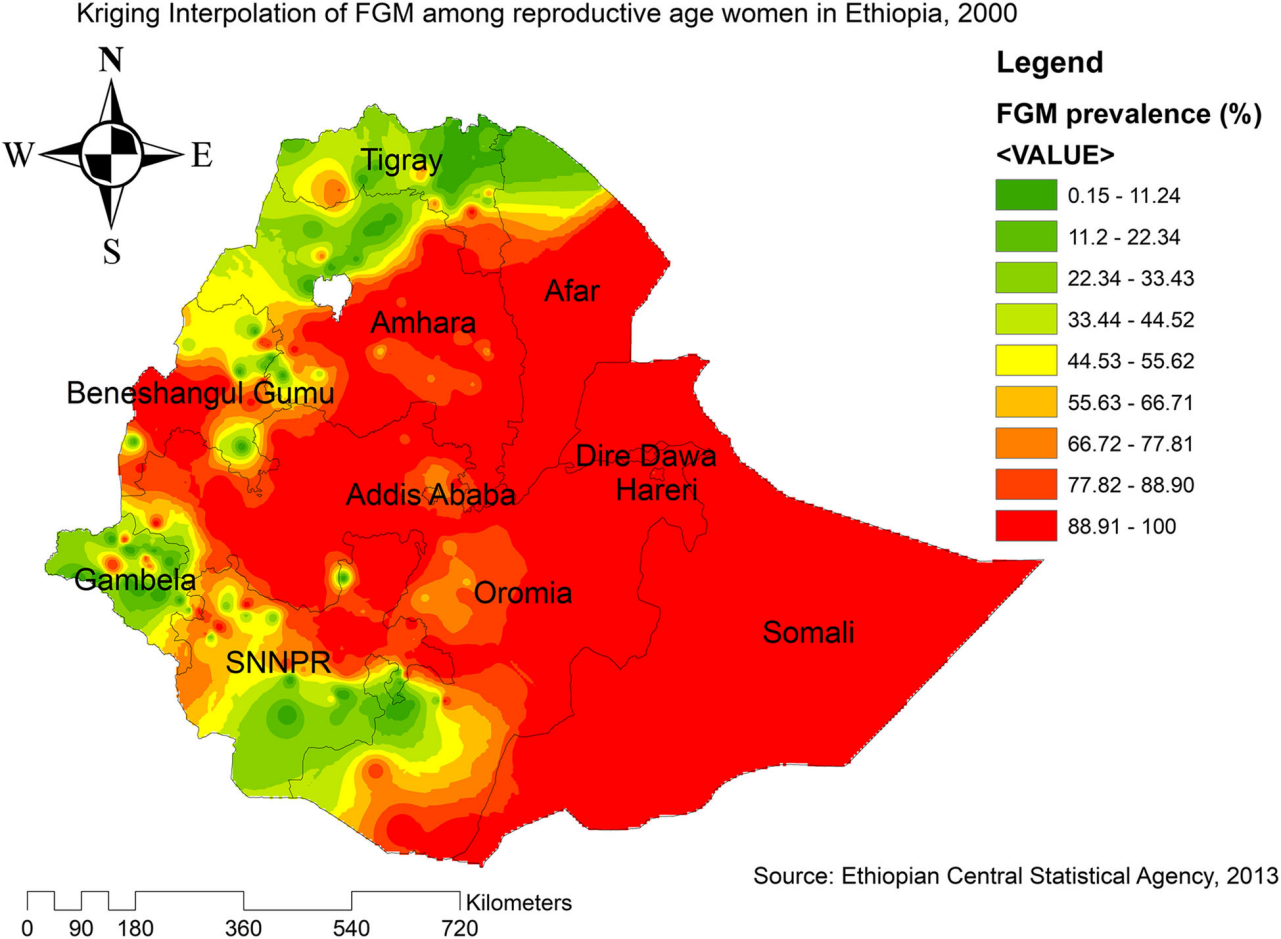
Interpolation of FGM practice in Ethiopia, 2000 (Source; Shape file from CSA, 2013, done using ArcGIS version 10.6 and SaTScan version 9.6)

**Fig. 8:**
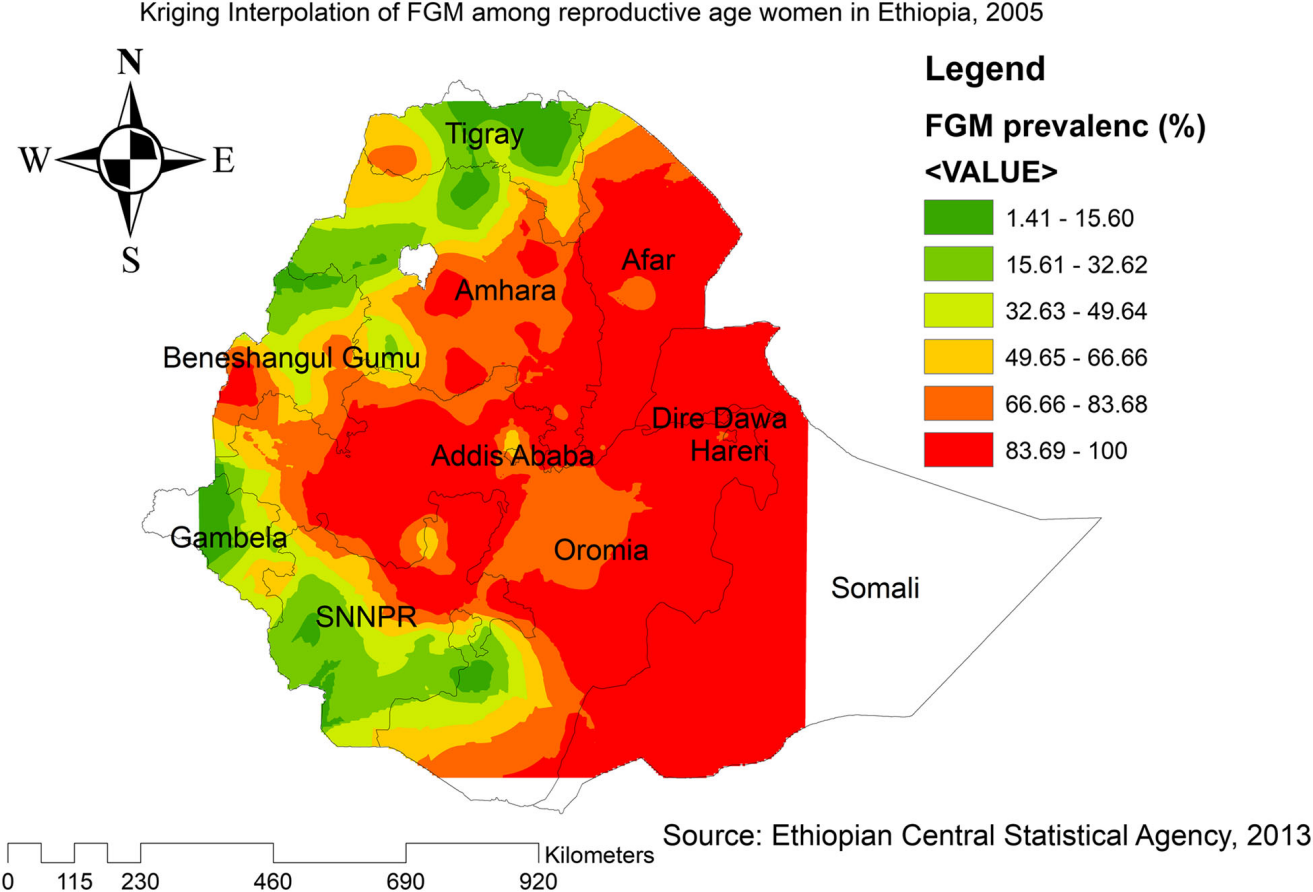
Interpolation of FGM practice in Ethiopia, 2005 (Source; Shape file from CSA, 2013, done using ArcGIS version 10.6 and SaTScan version 9.6)

**Fig. 9:**
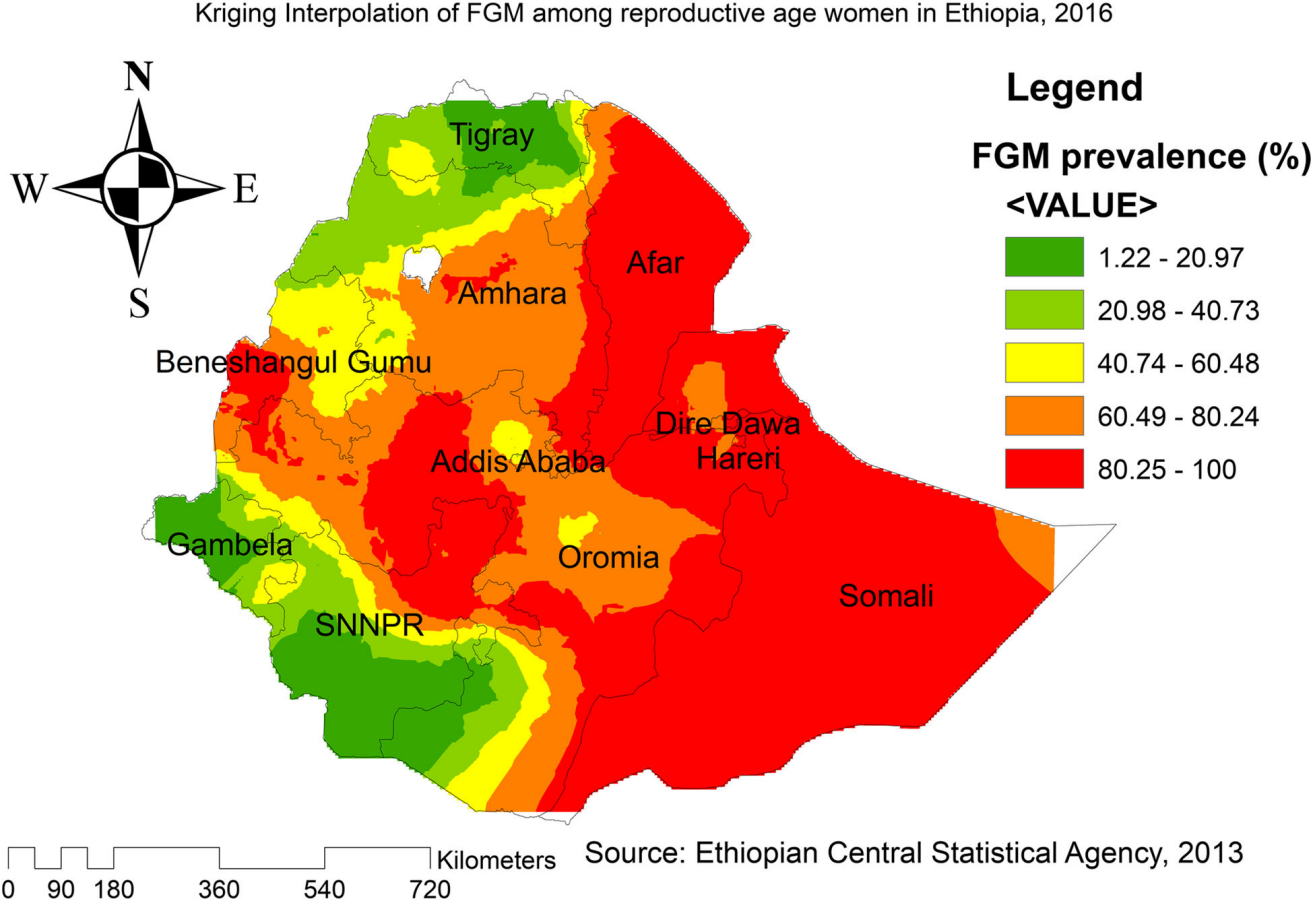
Interpolation of FGM practice In Ethiopia, 2016 (Source; Shape file from CSA, 2013, done using ArcGIS version 10.6 and SaTScan version 9.6)

Based on EDHS 2000 sampled data, Kriging interpolation predict that the highest prevalence of FGM was detected in the entire Somali, Oromia, Harari, Dire Dawa, Addis Ababa, Benishangul Gumuz, southeast Afar, southeast Amhara, and east SNNPRs. In contrast, relatively low prevalence of FGM was predicted in the entire Tigray, Gambella, southwest Oromia, north Benishangul, northwest Amhara, and north Afar (Fig. 7). Based on EDHS 2005, the highest prevalence of FGM was detected in east SNNPRs, Oromia, west Benishangul, southeast Amhara, and most of the Afar regions. In contrast, predicted low prevalence of FGM was detected in Tigray, northwest Gambella, north Afar, and west SNNPRs (Fig. 8). Based on EDHS 2016 data, Kriging interpolation predicted that most parts of Oromia, entire Somali, Dire Dawa, Harari, Afar, and west Benishangul contained the highest FGM prevalence while most parts of Amhara, Tigray, Gambella and west SNNPRs contained relatively low FGM practice (Fig. 9).

## Discussion

Despite the efforts to eliminate female genital mutilation globally [28], still, it remains a major public health problem in Africa including Ethiopia [29]. In this study, the prevalence of FGM.

in Ethiopia has decreased significantly over time. It is in line with studies done in Ghana [25], Oromia [30], Nigeria [31], Burkina Faso [32] and Gambia [33]. This could be due to the coordinated efforts of government and non-governmental organizations to eliminate FGM by engaging the community to speed up the FGM practice’s abandonment [34].

The prevalence of FGM has decreased over time from 79.9 to 70.4% from 2000 to 2016 with an annual rate of reduction of 0.8%. While the incidence of FGM has been decreased, the decline rate is far below the Sustainable Development Goal target 5 and study findings in Eastern Africa (3.2%), West Africa (2.3%) and North Africa (1.9%) [34]. The possible explanation could be because FGM is a deeply rooted cultural practice in Ethiopia that has persisted as a social norm for a long time [35]. This implies that advanced public health programs targeting FGM abandonment should be done to achieve the SDG goals.

The multivariate decomposition analysis identified the significant factors that contributed to the decrease in FGM practice over the last 16 years. The overall decomposition analysis revealed that about 95% of the decrease in FGM over the past 16 years was explained by the difference in the effects of characteristics (coefficient) between the surveys. About 20.6% of the decrease in FGM practice in Ethiopia was due to the difference in FGM practice among the urban population. This is in line with a study done in Egypt [36]. This could be due to improved access to information and education over time that could help urban residents become more aware of the negative health consequences of FGM [37].

The difference in the effect of Muslim and orthodox religious followers significantly contributed to the decrease in FGM practice over time, which was supported by the previous study [38]. This showed that the behavior of religious followers towards FGM practice has been improved over time. The change could be due to the engagement of faith leaders by the government to improve awareness of the community towards the consequence of FGM in Ethiopia [34]. The other contributing factors for the change in FGM practice over the last 16 years were: the change in the effect of women’s occupation, women education, region (Tigray region), and media exposure. This could be due to the improvement in media exposure; job opportunity and access to education that significantly contribute to women empowerment and their capacity to fight against harmful traditional practices such as FGM [1, 39]. Therefore, women’s education, media exposure, and job opportunities significantly contributed to the decrease in FGM practice over time [40, 41].

The spatial analysis revealed that there was a significant Spatio-temporal variation of FGM practice across the country over time. This finding was supported by a spatial study done in Nigeria [42], and Kenya [43]. The hotspot and SaTScan analysis detected significant hotspot areas across the three EDHS surveys, which were consistently located in the entire Somali, Afar, Harari, and border areas of Somali regions. Even though there is a coordinated effort by engaging faith and community organizations to accelerate the abandonment of FGM practice in Ethiopia [44]; Afar, Harari, and Somali regions were significant hot spot areas where FGM was highly practiced. This could be due to deeply rooted beliefs, attitudes, and behaviors towards FGM practice in these regions [45]. Furthermore, the belief that if a girl not gets circumcised, the daughter may not get married, and this makes the community practice female circumcision to get social acceptance [46]. Besides, there is community resistance to abandoning FGM practice in Somali and Afar region [47]. This implies that the government needs to strengthen public health efforts to reduce the FGM practice in these regions by highly engaging the community leaders, civil society, school, media, faith leaders, and females.

### Strengths and limitations

This study had several strengths. First, the study was based on nationally representative large datasets, and thus it had adequate statistical power. Second, the estimates of the study were done after the data were weighted for probability sampling and non-response. Therefore, it can be generalized to all women in Ethiopia. Third, multivariate decomposition analysis was applied to identify the factors contributing to the decrease in FGM practice over time. Fourth, the use of GIS and SaTScan statistical tests helped to detect statistically significant hotspot areas of FGM practice across the surveys and to design effective public health programs.

As a limitation, some variables were not consistently collected in all EDHS surveys like the wealth index. Therefore, these variables were not used for the decomposition analysis. The other limitation was the SaTScan detect only circular clusters not irregularly shaped clusters. Furthermore, the EDHS survey did not incorporate community-level variables like community norm, culture, and beliefs rather it relied on mothers or caregivers report and might have the possibility of social desirability and recall bias since FGM is not socially acceptable though CSA claim that strong effort was made to minimize it mainly through extensive training of data collectors, recruiting experienced data collectors and supervisors this might underestimate our finding. As with other cross-sectional studies, the temporal relationship between exposure and outcome can’t be established. Besides, since we used repeated cross-sectional data (time series cross-sectional study) for the trend and Spatio-temporal analysis, the enumeration areas selected in each survey were different, and the data for each survey were collected in different population, this could result in under/overestimation of the prevalence of FGM practice hence there may be the migration of the population to different enumeration areas during the study period, this may dilute the difference in FGM practice across the surveys since the enumeration areas selected across the survey was not the same in EDHSs even if we have done Kriging Interpolation to predict the unobserved areas of the country over the surveys.

## Conclusion

Female genital mutilations among women have shown a dramatic decrease over the last 16 years. The multivariate decomposition analysis showed that 95% of the overall decrease in FGM practice over the last 16 years was due to the differences in the effect of characteristics (coefficient) between the 2000 and 2016 EDHS. Mainly the decrease in FGM practice was attributed to the difference in the effects of residence, religion, region, media exposure, occupational status, and women education. But, the change in FGM practice due to the difference in endowment or composition between the surveys was not significant.

This study identified significant Spatio-temporal clusters of FGM practice in Somali, Afar, Harari, and border areas of Somali regions consistently over the three surveys. These results provide further insight into identifying the reason why FGM practice highly concentrated in these regions consistently over the three surveys and dig out the deeply rooted factors about the continued experience of FGM practice. Besides, it would help policymakers, programmers, and NGOs to design effective public health interventions and enabling efficient and timely spatial targeting to identify significant hotspot areas to achieve the SDGs.

## Data Availability

Data is available online and you can access it from www.measuredhs.com.

## Abbreviations

CI: Confidence Interval
CSA: Central Statistical Agency
DHS: Demographic Health Survey
EA: Enumeration Area
EDHS: Ethiopian Demographic Health Survey
FGM: Female Genital Mutilation
GDP: Gross Domestic Product
GIS: Geographic Information System
LLR: log-likelihood Ratio
LR: Likelihood Ratio
PHC: Population and Housing census
SNNPRs: Southern Nations and Nationality People Regional state
WHO: World Health Organization
